# Dietary carbohydrates impair the protective effect of protein restriction against diabetes in NZO mice used as a model of type 2 diabetes

**DOI:** 10.1007/s00125-018-4595-1

**Published:** 2018-03-17

**Authors:** Thomas Laeger, Teresa Castaño-Martinez, Martin W. Werno, Lukasz Japtok, Christian Baumeier, Wenke Jonas, Burkhard Kleuser, Annette Schürmann

**Affiliations:** 1 Department of Experimental Diabetology, German Institute of Human Nutrition Potsdam-Rehbruecke (DIfE), Arthur-Scheunert-Allee 114-116, 14558 Nuthetal, Germany; 2 German Center for Diabetes Research (DZD), München-Neuherberg, Germany; 3 Department of Toxicology, Institute of Nutritional Science, University of Potsdam, Potsdam, Germany; 4 Institute of Nutritional Science, University of Potsdam, Nuthetal, Germany

**Keywords:** Energy expenditure, FGF21, Hyperglycaemia, Insulin resistance, NZO, Obesity, Protein restriction

## Abstract

**Aims/hypothesis:**

Low-protein diets are well known to improve glucose tolerance and increase energy expenditure. Increases in circulating fibroblast growth factor 21 (FGF21) have been implicated as a potential underlying mechanism.

**Methods:**

We aimed to test whether low-protein diets in the context of a high-carbohydrate or high-fat regimen would also protect against type 2 diabetes in New Zealand Obese (NZO) mice used as a model of polygenetic obesity and type 2 diabetes. Mice were placed on high-fat diets that provided protein at control (16 kJ%; CON) or low (4 kJ%; low-protein/high-carbohydrate [LP/HC] or low-protein/high-fat [LP/HF]) levels.

**Results:**

Protein restriction prevented the onset of hyperglycaemia and beta cell loss despite increased food intake and fat mass. The effect was seen only under conditions of a lower carbohydrate/fat ratio (LP/HF). When the carbohydrate/fat ratio was high (LP/HC), mice developed type 2 diabetes despite the robustly elevated hepatic FGF21 secretion and increased energy expenditure.

**Conclusion/interpretation:**

Prevention of type 2 diabetes through protein restriction, without lowering food intake and body fat mass, is compromised by high dietary carbohydrates. Increased FGF21 levels and elevated energy expenditure do not protect against hyperglycaemia and type 2 diabetes per se.

**Electronic supplementary material:**

The online version of this article (10.1007/s00125-018-4595-1) contains peer-reviewed but unedited supplementary material, which is available to authorised users.

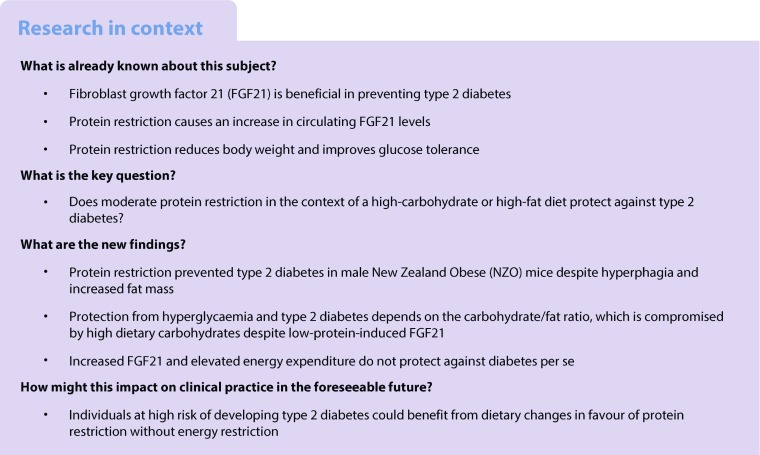



## Introduction

Energy restriction (e.g. caloric restriction, intermittent fasting) has a positive effect on metabolic health, improving insulin sensitivity and preventing obesity and type 2 diabetes. Dietary protein restriction is an emerging alternative for treating obesity and glucose intolerance induced by a high-fat diet [1, 2]. Low-protein diets reduce body weight by decreasing body fat gain, improving glucose tolerance and increasing energy expenditure. These effects are mediated by fibroblast growth factor 21 (FGF21) [3–6].

Circulating FGF21, mainly produced by the liver, is also expressed in the thymus, gut, brain, adipose tissue, muscle and pancreas [3, 4, 7]. FGF21 targets organs through a cell-surface receptor complex composed of the traditional FGF receptor, FGFR1c, and the necessary FGF co-receptor β-Klotho [8]. Administration of FGF21 to mice induces activation of brown adipose tissue (BAT), and increases energy expenditure and insulin sensitivity [9–11]. The nervous system is the direct target of FGF21, whereas β-Klotho in adipose tissue and liver is dispensable for FGF21 effects on weight loss [12]. Furthermore, FGF21 improves beta cell function and survival [13], and prevents pancreatic inflammation [14]. This makes FGF21 a novel target for treating diabetes.

FGF21 levels correlate positively with blood glucose levels, as has been shown in hyperglycaemic and obese New Zealand Obese (NZO) mice used as a model of diabetes, in which plasma FGF21 levels reach approximately 0.8 ng/ml [11]. This increase in FGF21 concentration is considered to be a compensatory mechanism for the worsening of glucose and lipid metabolism. Dietary protein restriction increases circulating FGF21 levels to above 4 ng/ml in rats and BL6 mice after 4 days on the diet [3, 4, 6]. Exogenous FGF21 treatment improves glucose homeostasis and prevents hyperglycaemia and diabetes in NZO mice, a model of polygenetic obesity and type 2 diabetes with the characteristic trait of pancreatic beta cell loss [11, 15]. We concluded that the diabetes-susceptible NZO mouse is not FGF21-resistant, and is a potential animal model to study dietary low-protein-triggered, FGF21-dependent outcomes related to diabetes prevention. Therefore, in the present study, we tested whether moderate protein restriction in a high-carbohydrate or high-fat regimen would protect against diabetes in NZO mice.

## Methods

### Animals, diets, and experimental design

**Fig. 1:**
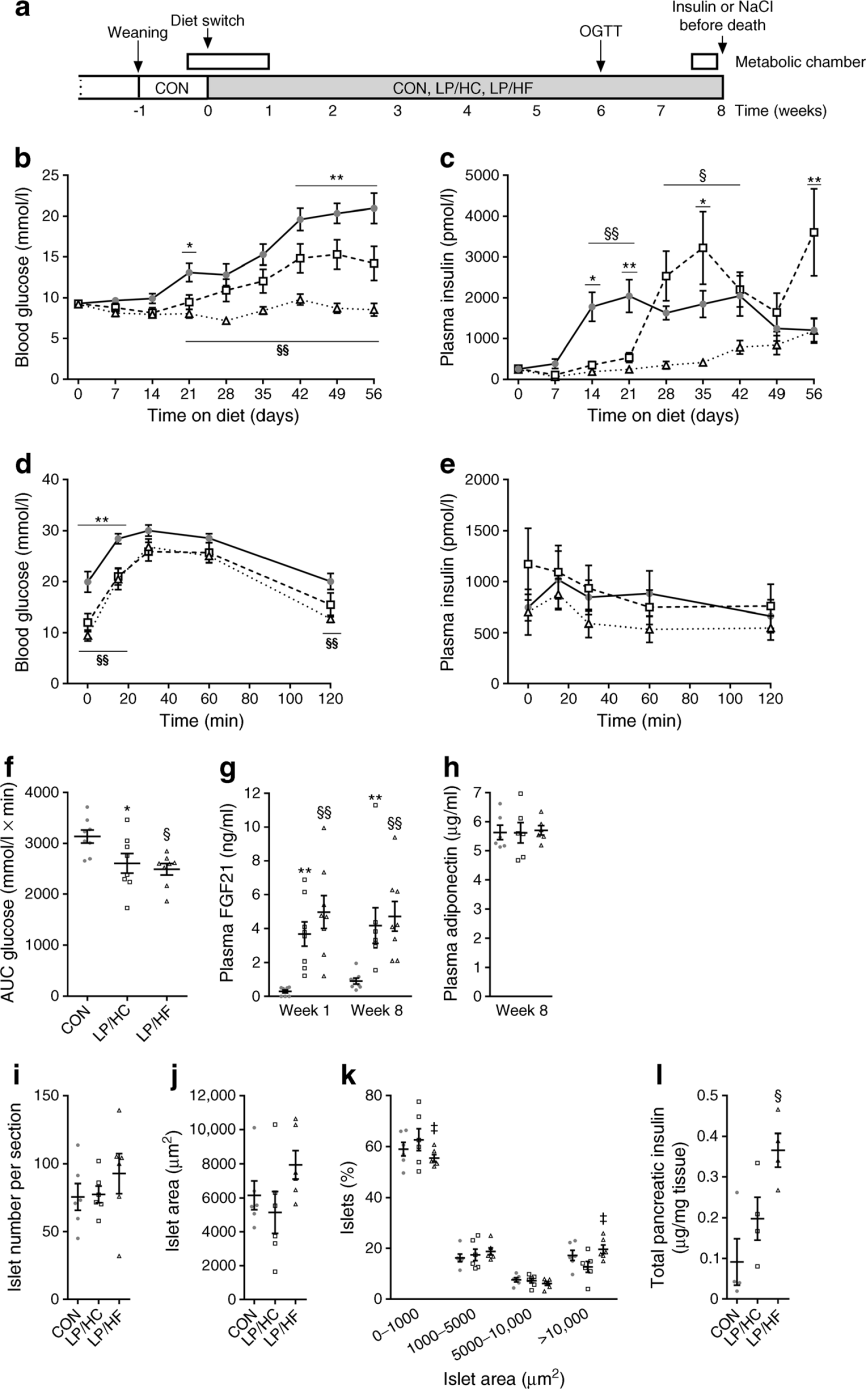
Prevention of hyperglycaemia by protein restriction is impaired by high dietary carbohydrates. (**a**) Study design. At 3 weeks of age, NZO mice were placed on the CON diet for 1 week, at which point a random subgroup of animals was transferred to the LP/HC or LP/HF diet for 8 weeks. (**b**) Random blood glucose and (**c**) plasma insulin concentrations. Six weeks after the dietary switch, an OGTT (glucose 2 mg/g body weight by oral gavage) was performed in mice fasted for 2 h. (**d**) Blood glucose, (**e**) plasma insulin, and (**f**) AUC of glucose levels during the OGTTs. Circulating (**g**) FGF21 and (**h**) adiponectin levels assessed by ELISA in NZO mice consuming control or LP diets for the indicated time (*n* = 6–8 per group). (**i**) Pancreatic islet number, (**j**) islet area, and (**k**) islet area distribution in mice at the end of the study (*n* = 6 per group). (**l**) Total pancreatic insulin (*n* = 4 per group). Grey circles, CON; white squares, LP/HC; white triangles, LP/HF. Data are presented as means ± SEM (*n* = 8–16 per group). Differences vs the CON group were calculated by two-way ANOVA (**b**–**e**) and one-way ANOVA (**f**–**l**). *p < 0.05, **p < 0.01, CON vs LP/HC; ^§^*p* < 0.05, ^§§^*p* < 0.01, CON vs LP/HF; ^‡^*p* < 0.05, LP/HC vs LP/HF

NZO/HIBomDife mice (German Institute of Human Nutrition Potsdam-Rehbruecke [DIfE], Nuthetal, Germany) were housed singly under 12 h light/12 h dark cycle (lights on at 06:00 h) at a temperature of 21 ± 1°C with ad libitum access to food and water unless otherwise noted. At 3 weeks of age, male NZO mice were placed on a control (CON) diet (S8022-E122, ssniff, Soest, Germany; electronic supplementary material [ESM] Table 1) for 1 week, at which point a subgroup of animals was transferred to a low-protein/high-carbohydrate (LP/HC; S8022-E120) or low-protein/high-fat (LP/HF; S8022-E121) diet for 8 weeks (Fig. 1a).

Six weeks after switching diets, an OGTT was performed after a 2 h period of fasting (glucose 2 mg/g body weight). At the indicated time points, blood glucose and plasma insulin levels were measured. Body weight, food intake, body composition (quantitative magnetic resonance; EchoMRI 2012 Body Composition 115 Analyzer; Houston, TX, USA), random blood glucose and equivalent serum insulin levels were measured weekly in tail blood. For analysis of energy expenditure, transition to the dietary treatments occurred within metabolic chambers (PhenoMaster/LabMaster; TSE Systems, Bad Homburg, Germany). Eight weeks after switching diet, the mice were killed during mid-light cycle in a 6 h fasted state using acute exposure to isoflurane; this was followed by blood collection. Mice were treated subcutaneously with NaCl (0.9% wt./vol.) or insulin (7 U/kg body weight) 15 min before killing, and tissues were collected. Blood was centrifuged at 10,000 *g* at 4°C for 10 min. Tissues were collected and snap-frozen in liquid nitrogen for further analysis. All procedures involving animals were approved by the animal welfare committees of DIfE and local authorities (Landesamt für Umwelt, Gesundheit und Verbraucherschutz, Brandenburg, Germany).

### Determination of adiponectin, FGF21, and insulin

Plasma adiponectin, FGF21 and insulin concentrations were determined by specific ELISAs (ESM Table 2) [3, 4, 11].

### Pancreatic insulin content

For the pancreatic insulin analysis, the entire pancreas was homogenised in ice-cold acidic ethanol (0.1 mol/l HCl in 70% vol./vol. ethanol) and incubated for 24 h at 4°C. After centrifugation (16,000 *g*, 10 min), insulin was detected by ELISA (ESM Table 2).

### Western immunoblot analysis

Western blot analysis was performed as previously described [16] using 20 μg protein/sample solutions (see ESM Table 2 for a list of antibodies used). Experimental controls were used to validate antibodies.

### Immunohistochemistry and morphometric analysis of pancreatic islets

Three longitudinal serial sections of pancreas tissue per animal (6 μm thickness, sampling intervals 140 μm) were prepared for insulin staining (ESM Table 2) as previously described [17], and were analysed.

### Detection of liver triacylglycerol and glycogen concentrations

Hepatic triacylglycerol content was measured using the commercial TR-210 kit (Randox, Crumlin, UK). Quantification of hepatic glycogen content was performed as previously described [11].

### Detection of plasma triacylglycerol and NEFA

Plasma triacylglycerol content was measured using the commercial TR0100 kit (Sigma-Aldrich, Munich, Germany). Plasma NEFA levels were enzymatically analysed using the NEFA-HR(2) assay (Wako Chemicals, Neuss, Germany).

### Mass spectrometry of hepatic lipids

Ceramides, sphingomyelin and diacylglycerols (DAGs) were extracted as previously described [18]. Briefly, lipid extraction was performed using C17-ceramide, C16-d_31_-sphingomyelin, 1,3-dipentadecanoin (C15:0/C15:0 DAG) and 1,3-diheptadecanoin-d_5_ (C17:0/C17:0-d_5_ DAG) as internal standards. A saponification step applied for extraction of ceramides and sphingomyelins was omitted for DAG extraction. Analyses were conducted using a 1200 series HPLC coupled to a Q-TOF 6530 mass spectrometer (Agilent Technologies, Santa Clara, CA, USA) operating in positive ESI mode. Ceramides and sphingomyelins were analysed in MS/MS mode using fragmentation of precursor ions into the product ions *m*/*z* 264.270 and *m*/*z* 184.074, respectively [18]. DAG were analysed in MS mode monitoring following [M+Na]^+^ ions: C15:0/C15:0 DAG (*m*/*z* 563.465), C32:0 DAG (*m*/*z* 591.496), C34:2 DAG (*m*/*z* 615.496), C16:0/C18:1 DAG (*m*/*z* 617.512), C17:0/C17:0-d5 DAG (*m*/*z* 624.559), C36:4 DAG (*m*/*z* 639.496), C36:3 DAG (*m*/*z* 641.512) and C36:2 DAG (*m*/*z* 643.527). Quantification was performed with MassHunter software (Agilent Technologies; version B.06.00).

### Real-time PCR

RNA extraction from liver, gonadal white adipose tissue (gWAT), subcutaneous white adipose tissue (sWAT) and BAT, and real-time PCR were conducted as previously described [11]. Target gene expression (*Acc1*, *Cidea*, *Dio2*, *Fasn*, *Fgf21*, *Glut1* [also known as *Slc2a1*], *Glut4* [also known as *Slc2a4*], *Klb*, *Prdm16*, *Scd1*, *Ucp1*) was normalised to cyclophilin A (*Ppia*) as an endogenous control.

### Statistical analysis

Data were analysed using software Prism 6 (GraphPad Software, San Diego, CA, USA) applying one-way ANOVA, two-way ANOVA or unpaired two-tailed *t* test. Energy expenditure analysis with body weight as covariate was assessed via ANCOVA using the MMPC.org ANCOVA data analysis tool (https://www.mmpc.org/shared/regression.aspx; accessed 1 March 2017). All data are expressed as means ± SEM, with a probability value of 0.05 considered statistically significant. Samples were randomised and no data were omitted. The experimenters were not blind to group assignment.

## Results

### Prevention of hyperglycaemia by protein restriction is impaired by high dietary carbohydrates

To test whether protein restriction would protect against glucose intolerance and diabetes induced by a high-fat diet, 3-week-old male NZO mice were placed on a CON diet (16 kJ% protein) for 1 week, at which point a random subgroup of animals was transferred to the LP/HC (4 kJ% protein, 63 kJ% carbohydrate) or LP/HF (4 kJ% protein, 47 kJ% fat) diet for 8 weeks (Fig. 1a). CON mice exhibited a steady rise of blood glucose until the end of the study (Fig. 1b; blood glucose at week 8 = 21.0 ± 1.8 mmol/l). In contrast, LP/HF mice displayed normal blood glucose levels throughout the study (blood glucose at week 8 = 8.5 ± 0.8 mmol/l), whereas LP/HC mice showed a delay of 2 weeks in the increase in blood glucose levels compared with CON mice (Fig. 1b; blood glucose at week 8 = 14.2 ± 2.1 mmol/l). Whereas plasma insulin levels rose rapidly at the age of 6 weeks in CON mice, insulin levels began to increase robustly 2 weeks later in LP/HC mice (Fig. 1c, ESM Fig. 1). LP/HF mice, however, displayed normal plasma insulin levels that tended to increase slowly until the end of the study (Fig. 1c). An OGTT conducted after the mice had been on the low-protein diets for 6 weeks indicated that LP/HC and LP/HF improved glucose clearance relative to CON mice (Fig. 1d, f). Insulin levels during the OGTTs did not differ between the groups (Fig. 1e). As expected, consumption of the LP/HC and LP/HF diets markedly increased plasma FGF21 concentrations after either 1 or 8 weeks (Fig. 1g). To investigate the source of the increased FGF21, we measured *Fgf21* mRNA expression in liver, gWAT, sWAT and BAT after 8 weeks on the low-protein diets. Whereas hepatic *Fgf21* mRNA expression was significantly increased, there was no increase in *Fgf21* mRNA expression in gWAT, sWAT or BAT (ESM Fig. 2a). Confirming earlier studies [3, 5], these data indicate that dietary protein restriction is a potent stimulator of hepatic and circulating FGF21.

As indicated in Fig. 1b, only LP/HF mice are protected against hyperglycaemia, whereas LP/HC mice showed an increased blood glucose, but both LP/HF and LP/HC mice exhibited increased circulating levels of FGF21 (Fig. 1g). We therefore asked what signal might impair FGF21 action. mRNA expression of the FGF21 co-receptor β-Klotho (*Klb*) was not affected in liver, gWAT, sWAT and BAT (ESM Fig. 2b). Adiponectin mediates the metabolic effects of FGF21 on insulin sensitivity and glucose homeostasis, but plasma adiponectin concentrations did not differ between the groups (Fig. 1h). Histological analysis of pancreatic islets at the end of the study revealed a higher number of islets and a larger islet area in LP/HF mice (although this was not significant; Fig. 1i, j). Additionally, the number of islets over 10,000 μm^2^ in size was significantly increased, and the number of islets less than 1000 μm^2^ was reduced in LP/HF mice compared with LP/HC mice (Fig. 1k). This might explain the significantly increased pancreatic insulin content in LP/HF mice (Fig. 1l), demonstrating that protein restriction might protect against beta cell loss in LP/HF mice. In summary, prevention of hyperglycaemia through protein restriction is compromised by high dietary carbohydrates despite increased FGF21 levels.

### Protein restriction induces hyperphagia and weight gain by increasing body fat mass

**Fig. 2:**
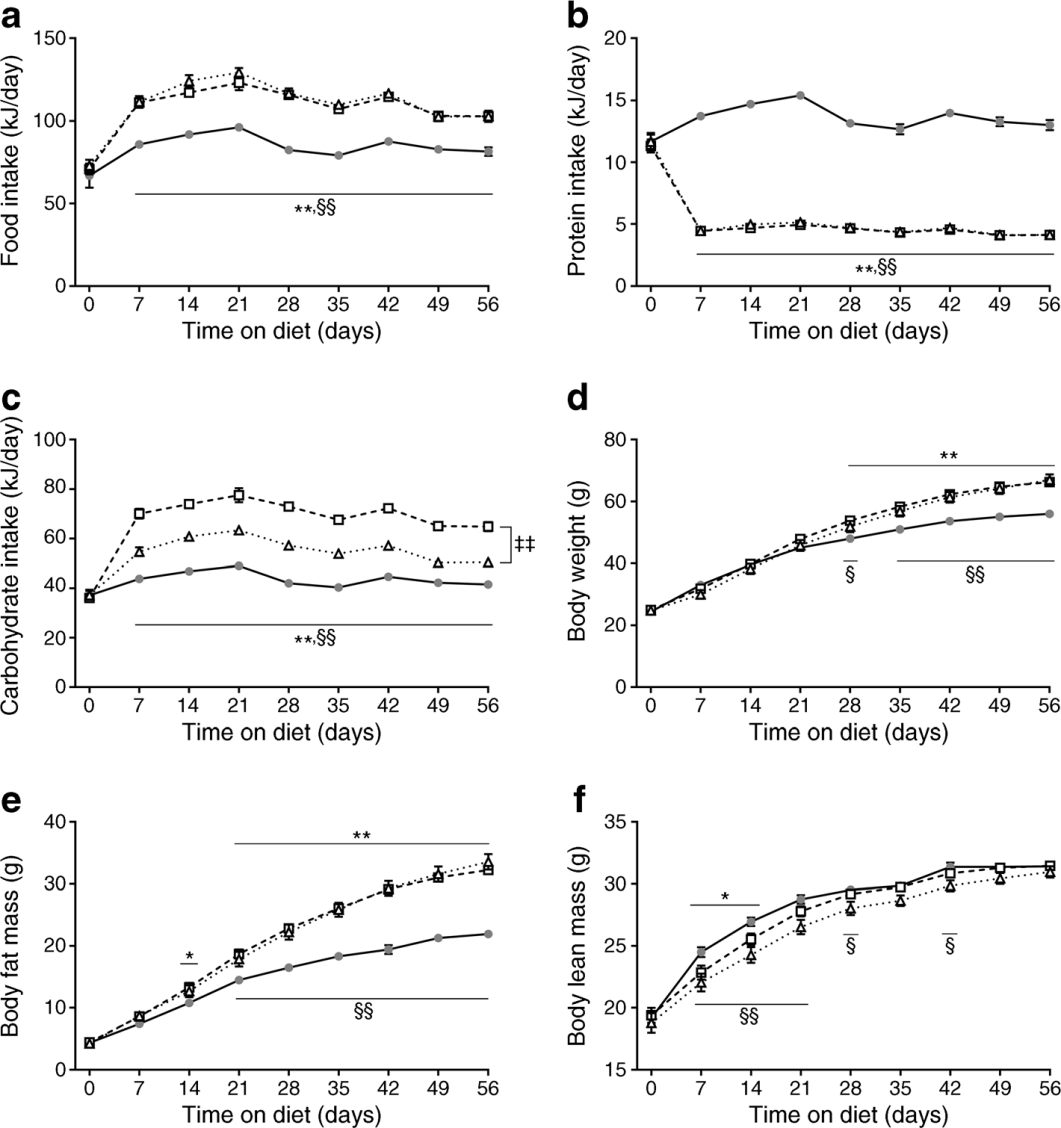
Dietary protein restriction induces hyperphagia and weight gain by increasing body fat mass in young NZO mice. Mice were treated as described in Fig. 1. (**a**) Food intake, (**b**) protein intake, (**c**) carbohydrate intake, (**d**) body weight, (**e**) body fat mass, and (**f**) body lean mass were monitored throughout the study. Grey circles, CON; white squares, LP/HC; white triangles, LP/HF. Data are presented as means ± SEM (*n* = 16 per group). Differences vs the CON group were calculated by two-way ANOVA. *p < 0.05, **p < 0.01, CON vs LP/HC; ^§^*p* < 0.05, ^§§^*p* < 0.01, CON vs LP/HF; ^‡‡^*p* < 0.01, LP/HC vs LP/HF

As expected, LP/HF and LP/HC mice showed an increased energy intake compared with CON mice (Fig. 2a), and this was caused by an increased food intake during the light period (ESM Fig. 3a). At the beginning of the light period, LP/HF mice displayed the highest food intake of all three groups, indicating that the low blood glucose measured at 07:00 h in LP/HF mice was not a prandial effect (ESM Fig. 3a). Calculations of macronutrient intake revealed that protein intake was significantly diminished in LP/HF and LP/HC mice (Fig. 2b). LP/HF mice with normal blood glucose levels displayed an increased intake of carbohydrates compared with CON mice (Fig. 2c). LP/HC mice showed an even higher consumption of carbohydrates than LP/HF mice (Fig. 2c). Unexpectedly, mice fed the low-protein diets gained more weight than CON mice due to a higher fat mass gain (Fig. 2d, e). In contrast, the lean mass was significantly lower at the beginning of the study in the mice receiving the low-protein diets, presumably due to muscle breakdown, although the groups did not differ in the last 2 weeks of the study (Fig. 2f). Collectively, these data demonstrate that the protection from hyperglycaemia by protein restriction is not driven by a reduction of body fat but limited to a specific amount of carbohydrate intake.

### Protein restriction increases energy expenditure

Earlier studies showed that FGF21 is required for low-protein-induced changes in energy expenditure, and that pharmacological FGF21 treatment acts in the brain and directly on adipose tissue to increase energy expenditure [3, 6, 12]. To test whether the energy expenditure might be different between the two low-protein groups despite a hyperphagic response, and explain the hyperglycaemia in LP/HC mice, energy expenditure was measured at the beginning and end of the study.

**Fig. 3:**
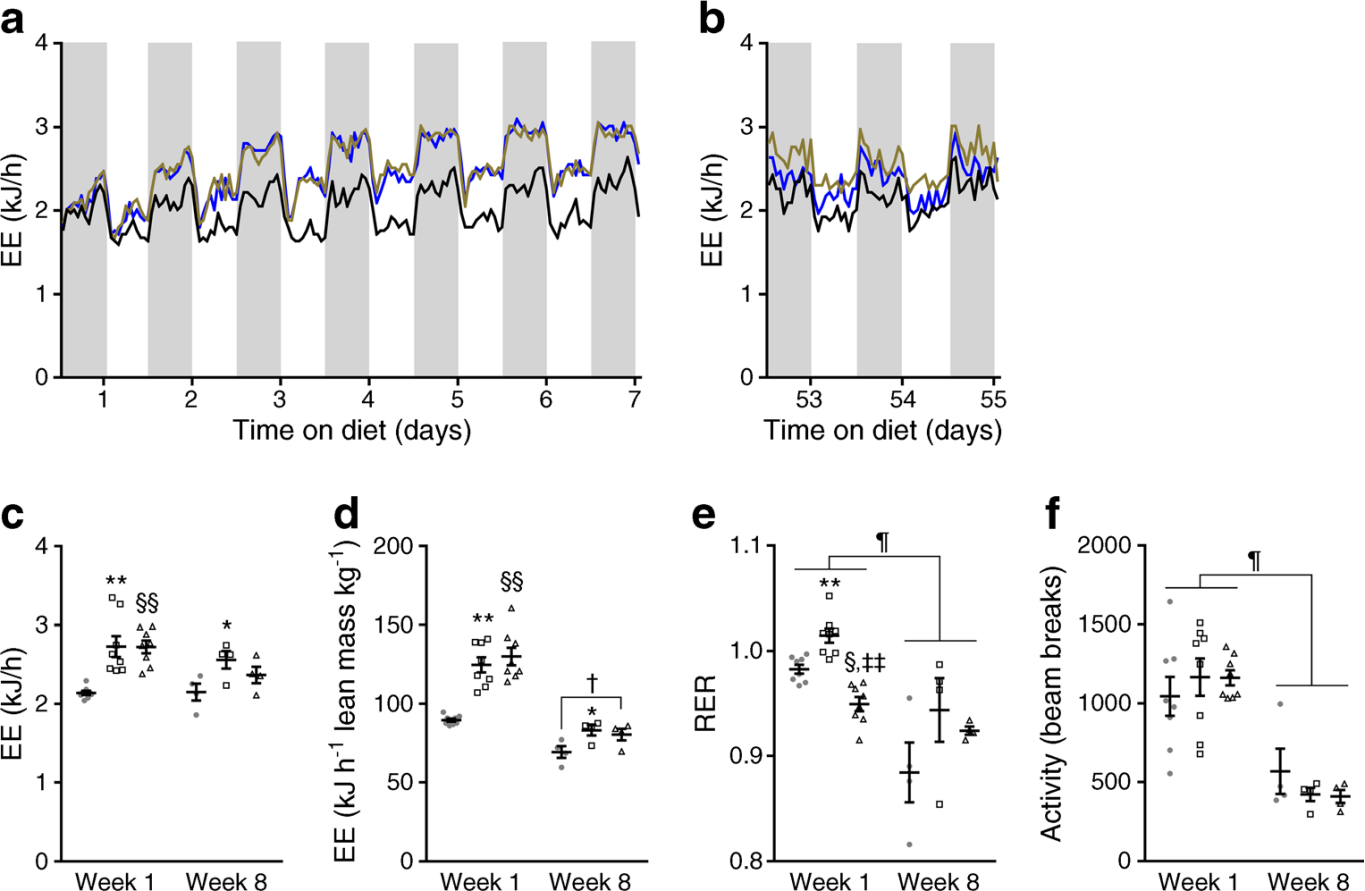
Protein restriction increases energy expenditure in NZO mice. Mice were treated as described in Fig. 1. (**a**) Energy expenditure (EE) in NZO mice consuming the CON or low-protein (LP/HC, LP/HF) diets for 1 week, and (**b**) after 7 weeks on the respective diet. (**c**) Average energy expenditure, (**d**) energy expenditure normalised to lean mass, (**e**) respiratory exchange ratio (RER), and (**f**) activity during week 1 (days 5–7) and week 8 (days 53–55) on the respective diet. Black line and circles, CON; brown line and squares, LP/HC; blue line and triangles, LP/HF. Data are presented as means ± SEM (week 1, *n* = 8 per group; week 8, *n* = 4 per group). Differences between groups were calculated by one-way ANOVA (**c**–**f**). *p < 0.05, **p < 0.01, CON vs LP/HC; ^§^*p* < 0.05, ^§§^*p* < 0.01, CON vs LP/HF; ^‡‡^*p* < 0.01, LP/HC vs LP/HF; ^¶^*p* < 0.05 as shown; † indicates non-significant difference, 0.1 > *p* > 0.05

As expected, LP/HF and LP/HC mice showed an increase in energy expenditure in the first week on the low-protein diet, beginning at day 2 after switching diets, an effect that was seen during both the dark and light periods (Fig. 3a). The increase in energy expenditure was observed irrespective of whether data were expressed on a per-animal basis (Fig. 3c) or normalised to lean mass (Fig. 3d). Energy expenditure analysis data using ANCOVA with body weight as the covariant demonstrated a low-protein-dependent increase in energy expenditure (ESM Fig. 3b). The respiratory exchange ratio was significantly increased by the LP/HC diet and decreased by the LP/HF diet, which reflects the expected changes in rate of carbohydrate and fatty acid oxidation, respectively (Fig. 3e). In contrast, there were no dietary effects on locomotor activity (Fig. 3f). Interestingly, after 7 weeks on the low-protein diets, energy expenditure was significantly higher in the LP/HC mice than the CON mice (Fig. 3b). After 7 weeks on the low-protein diet, an increase in energy expenditure was again observed, irrespective of whether energy expenditure data were expressed on a per-animal basis (Fig. 3c) or normalised to lean mass (Fig. 3d). Compared with the first week on the low-protein diet, the energy expenditure normalised to lean mass was in general lower, which was mirrored by the decreased activity of the mice at the end of the study. Finally, differences in energy expenditure between both the two low-protein groups could not explain the differences in development of diabetes. The LP/HC group showed elevated blood glucose levels despite increased energy expenditure.

### Protein restriction improves fat storage in adipose tissue, which prevents ectopic hepatic fat accumulation

**Fig. 4:**
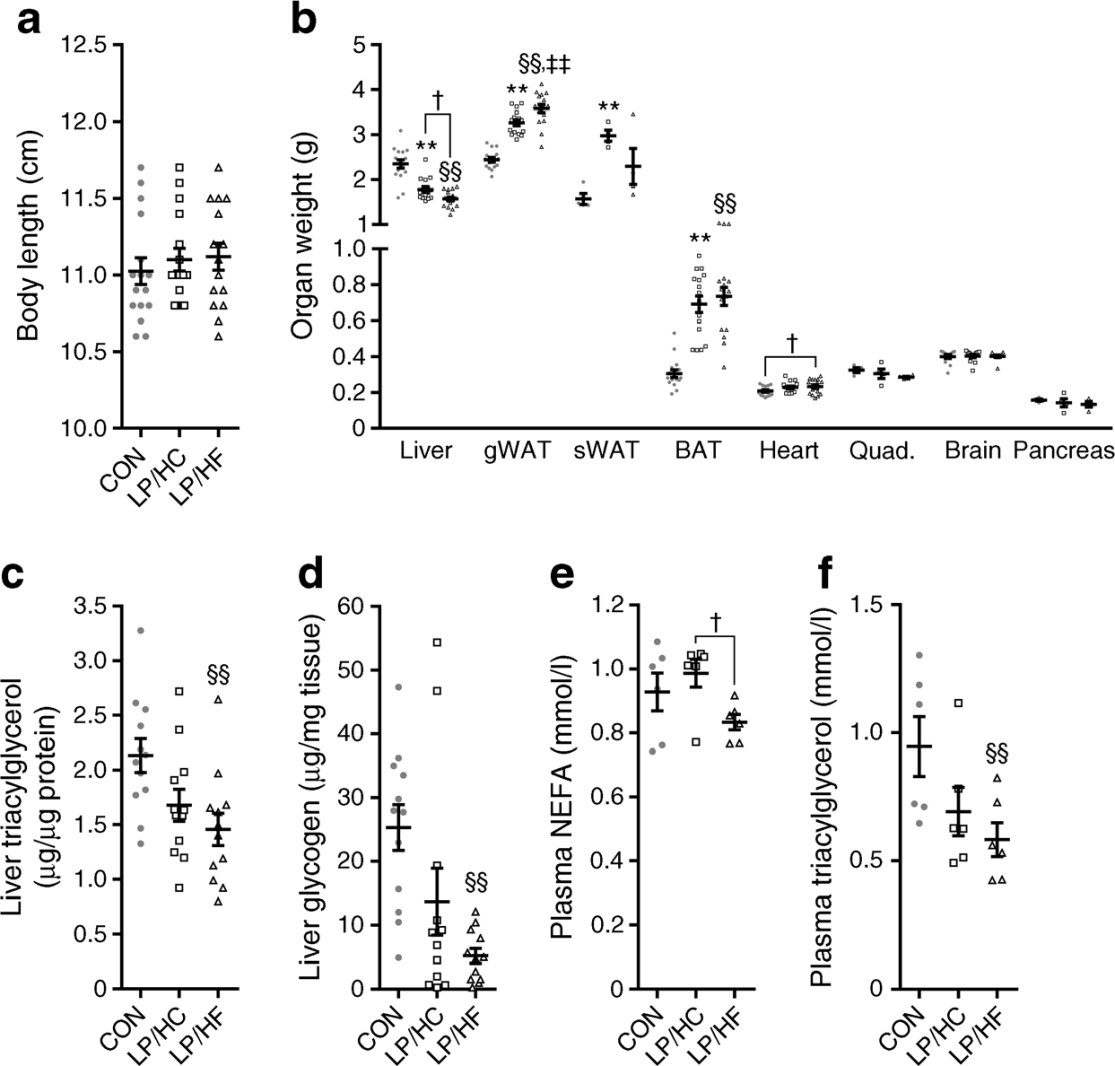
Protein restriction improves fat storage in adipose tissue, which prevents ectopic fat accumulation in the liver of young NZO mice. Mice were treated as described in Fig. 1. Eight weeks after the dietary switch, mice fasted for 6 h were killed. (**a**) Final body length. (**b**) Final weight of indicated organs. (**c**) Final liver triacylglycerol and (**d**) glycogen content. (**e**) Final plasma NEFA and (**f**) triacylglycerol concentrations. Grey circles, CON; white squares, LP/HC; white triangles, LP/HF. Quad., quadriceps. Data are presented as means ± SEM (*n* = 6–16 per group). Differences between groups were calculated by one-way ANOVA. **p < 0.01, CON vs LP/HC; ^§§^*p* < 0.01, CON vs LP/HF; ^‡‡^*p* < 0.01, LP/HC vs LP/HF; † indicates non-significant difference, 0.1 > *p* > 0.05

Interestingly, no effect on growth was observed throughout the study (Fig. 4a). However, final liver weight was significantly lower in the LP/HC and LP/HF than the CON mice (Fig. 4b), probably because of significantly decreased hepatic triacylglycerol (Fig. 4c) and glycogen (Fig. 4d) concentrations in LP/HF compared with CON mice. The former might explain the reduced NEFA and triacylglycerol concentrations in the circulation of LP/HF mice (Fig. 4e, f). In contrast to liver weight changes, gWAT, sWAT, and BAT mass were significantly higher in both groups of mice fed the low-protein diet (Fig. 4b). No differences could be observed between the groups in heart, quadriceps, brain and pancreas mass (Fig. 4b). In summary, protein restriction improves fat storage in adipose tissue, which prevents ectopic hepatic fat accumulation, and is more pronounced in the LP/HF than the LP/HC mice.

We then tested whether the robust increase in energy expenditure caused by the low-protein diets was associated with changes in thermogenic markers in BAT and sWAT, which is prone to browning. Unlike the acute induction caused by exogenous FGF21 [11], the sWAT thermogenic genes *Ucp1*, *Cidea* and *Prdm16* were not increased by low-protein-induced FGF21 (ESM Fig. 4a), as sWAT shows no evidence for browning under these conditions. Similarly, no difference in lipogenic genes (*Fasn*, *Scd1*, *Acc*) and genes for glucose uptake (*Glut1*, *Glut4*) could be measured in sWAT between the groups (ESM Fig. 4b). In contrast, the low-protein diets increased the mRNA expression of genes associated with lipogenesis within BAT (*Fasn*, *Scd1*, *Acc1*), whereas expression of *Glut1*, *Glut4*, *Ucp1* and *Cidea* was not induced (ESM Fig. 4c, d). These data demonstrate that low-protein-induced effects of FGF21 on thermogenesis are not detectable in NZO mice. It can be speculated that the increased BAT mass accounts for the increase in energy expenditure.

### Protein restriction alters hepatic lipid species

**Fig. 5:**
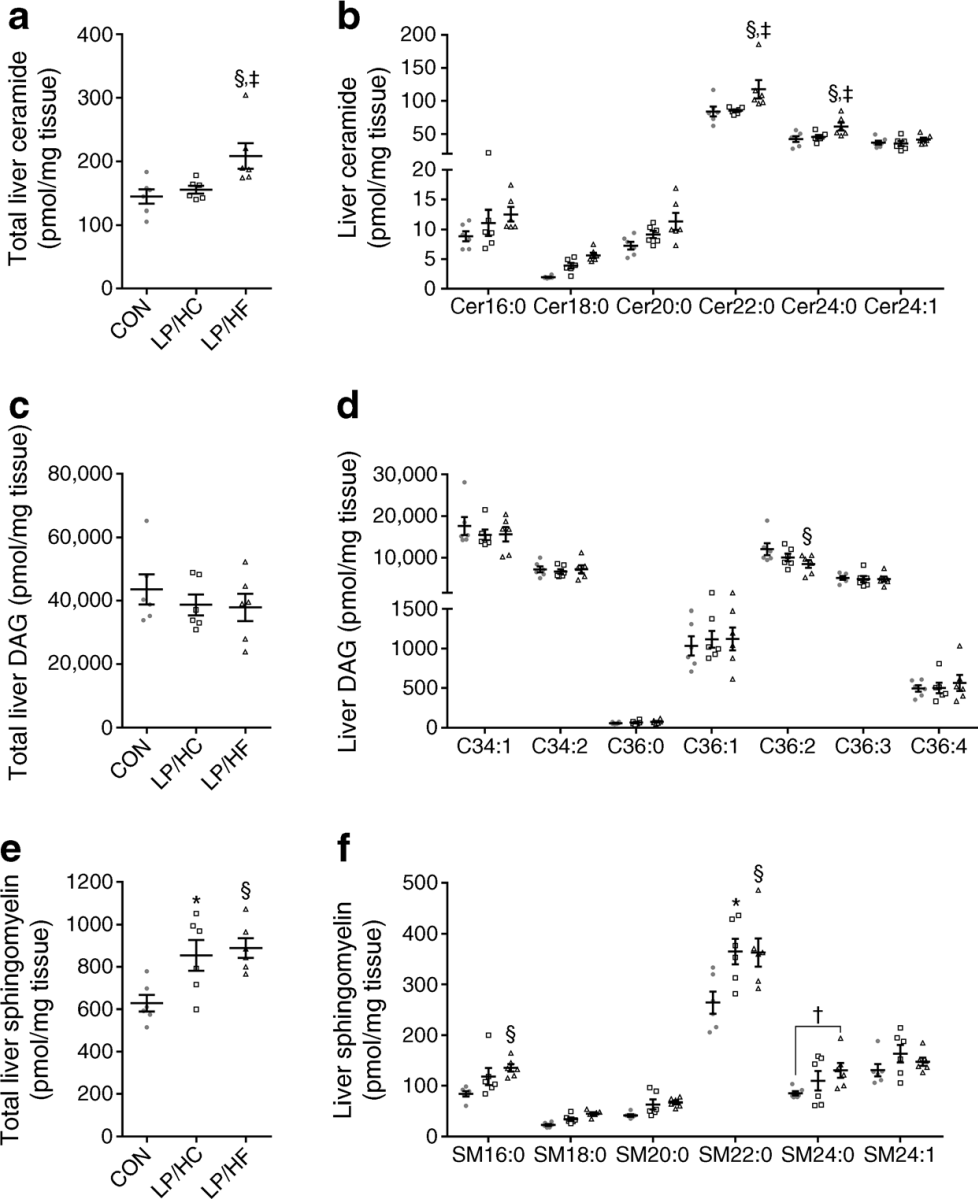
Dietary protein restriction alters hepatic lipid species in NZO mice. Mice were treated as described in Fig. 1. Eight weeks after the dietary switch, mice fasted for 6 h were killed. Total liver (**a**) ceramide content, (**b**) ceramide species, (**c**) total DAG content, (**d**) DAG species, (**e**) total sphingomyelin content, and (**f**) sphingomyelin species. Grey circles, CON; white squares, LP/HC; white triangles, LP/HF. Data are presented as means ± SEM (*n* = 6 per group). Differences between groups were calculated by one-way ANOVA. *p < 0.05, CON vs LP/HC; ^§^*p* < 0.05, CON vs LP/HF; ^‡^*p* < 0.05, LP/HC vs LP/HF; † indicates non-significant difference, 0.1 > *p* > 0.05

A growing number of studies have implicated ceramides, DAGs and sphingomyelins in insulin resistance [19–21]. We therefore extracted hepatic lipids and measured different lipid species. Significantly higher concentrations of total liver ceramides were seen in LP/HF compared with CON and LP/HC mice (Fig. 5a). As shown in Fig. 5b, the long-chain ceramides Cer22:0 and Cer24:0 were significantly increased in LP/HF compared with CON and LP/HC mice, whereas the shorter ceramides showed no difference in accumulation in the liver. No difference could be detected between groups in regard to total DAG content (Fig. 5c), except for the fact that C36:2 was significantly reduced in the liver of LP/HF compared with CON mice (Fig. 5d). Both groups of low-protein-fed mice showed a significant increase in total sphingomyelins compared with CON mice (Fig. 5e). The sphingomyelin SM16:0 was significantly increased in LP/HF compared with CON mice, and SM22:0 was significantly increased in both LP groups compared with CON mice (Fig. 5f). Taking these findings together, the lack of an increase in hepatic long-chain ceramides and the lack of reduced C36:2 in LP/HC mice might lead to impaired insulin sensitivity despite increased FGF21 levels.

### Protein restriction improves hepatic insulin sensitivity under conditions of a lower carbohydrate/fat ratio

**Fig. 6:**
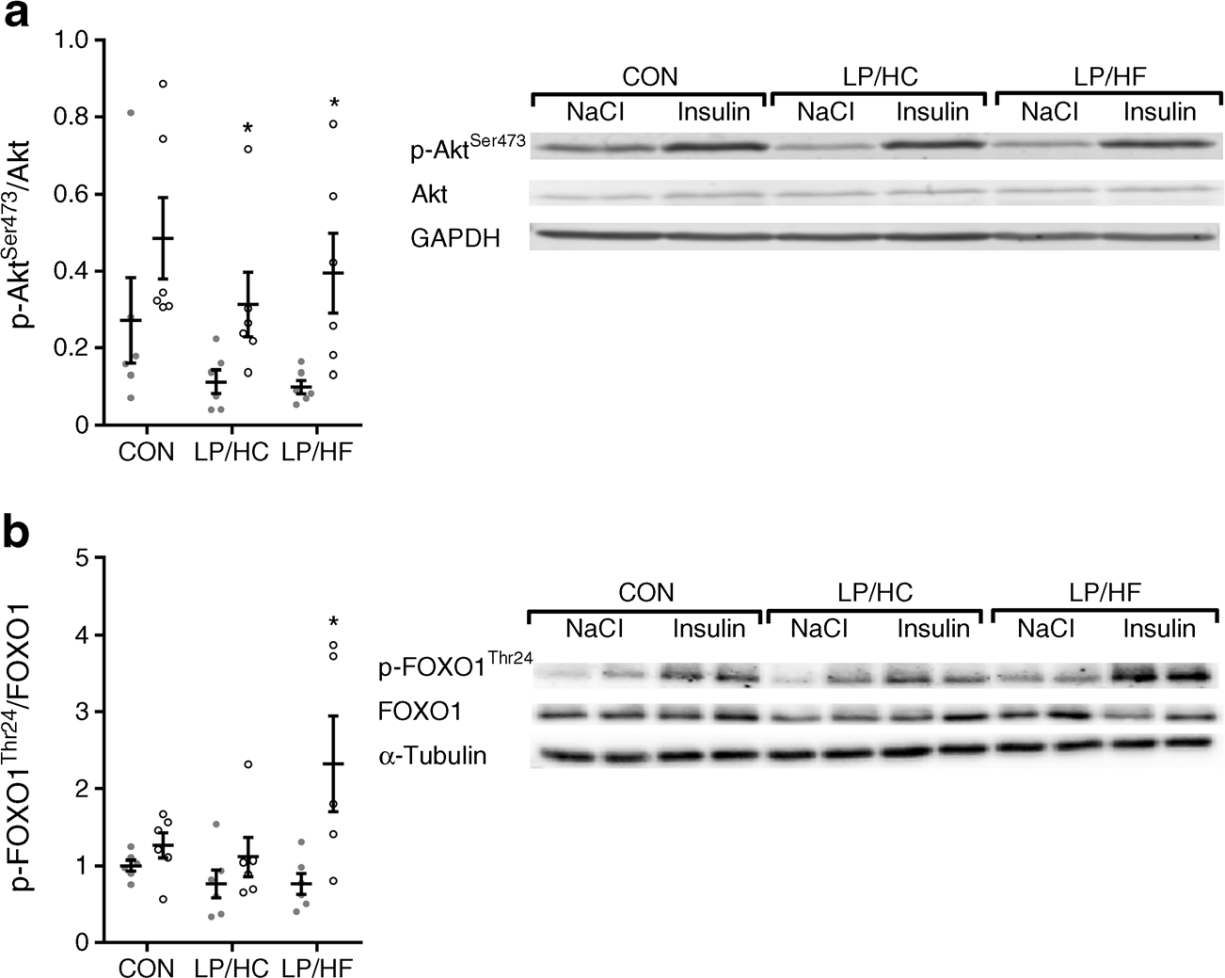
Dietary protein restriction slightly improves hepatic insulin sensitivity in NZO mice. Mice were treated as described in Fig. 1. Eight weeks after the dietary switch, mice fasted for 6 h were treated subcutaneously with NaCl or insulin (7 IU/BWkg) 15 min before killing. Western blots of total and phosphorylated (**a**) Akt and (**b**) FOXO1 in liver. Grey circles, NaCl; white circles, insulin. GAPDH, glyceraldehyde 3-phosphate dehydrogenase. Data are presented as means ± SEM (*n* = 6 per group). Differences between groups were analysed using a two-tailed *t* test. *p < 0.05

In order to test whether variations in long-chain ceramides and DAG associate with different insulin sensitivities and might explain the discrepancy in blood glucose levels between LP/HC (hyperglycaemic) and LP/HF (normoglycaemic) mice, all groups were treated with insulin or NaCl before killing, and the phosphorylation of Akt and forkhead box O1 (FOXO1), as read out from insulin signalling pathways, was measured. As shown in Fig. 6a, hepatic insulin sensitivity was slightly but significantly improved in LP/HC and LP/HF mice due to a lowering of basal Akt phosphorylation. In the quadriceps, insulin sensitivity was marginally improved by significant lowering of basal Akt phosphorylation in both low-protein-fed groups (ESM Fig. 5d). This was not the case in gWAT, sWAT, and BAT (ESM Fig. 5a–c). Strikingly, phosphorylation of FOXO1 was significantly increased in LP/HF mice (Fig. 6b). This might account for the reduced hepatic glycogen content (Fig. 4d) due to a reduced rate of gluconeogenesis, and might explain the divergence in blood glucose level between LP/HC and LP/HF mice.

## Discussion

In general, energy restriction is known to improve metabolic health [22, 23]. However, dietary protein restriction is an emerging alternative for treating obesity and glucose intolerance induced by a high-fat diet [1, 2]. Mediated by FGF21, dietary protein restriction reduces body weight gain, increases energy expenditure, changes food intake and metabolism, and improves glucose homeostasis in obese models [3–6].

Here we demonstrate for the first time that protein restriction prevents the onset of hyperglycaemia and beta cell loss in obese diabetes-susceptible NZO mice despite increased food intake and total fat mass. This depended on the carbohydrate/fat ratio in the diet. With a lower carbohydrate/fat ratio, NZO mice were protected from hyperglycaemia and beta cell loss, whereas under conditions of an increased carbohydrate/fat ratio, mice developed diabetes despite robustly elevated hepatic FGF21 secretion in response to dietary protein restriction. This is in contrast to the finding of improved glucose tolerance in NZO mice by Maida et al [1] under low-protein (5 kJ%) conditions, where the dietary carbohydrate content was even higher (85 kJ%). An explanation might be that the low fat content of the diet (10 kJ%) prevented the damage of beta cells by lipotoxicity in NZO mice. Unfortunately, the authors did not provide data on random blood glucose and insulin levels during their intervention with a low-protein diet [1].

In the current study, protein restriction in the context of a high-fat diet (LP/HF) protected against hyperglycaemia and beta cell loss, and improved insulin sensitivity. These effects occurred despite an increased energy intake, and might be explained by an FGF21-mediated increase in energy expenditure. LP/HF treatment also increased circulating FGF21 levels by approximately tenfold relative to CON, as a result of increased hepatic *Fgf21* mRNA expression. Therefore, these data correspond with the finding of FGF21 as an endocrine signal for a dietary protein restriction independent of energy restriction [3, 5]. As recently shown in NZO mice [11], exogenous FGF21 treatment induces thermogenic markers in sWAT, pointing towards a browning capacity in sWAT. In this study, despite increased FGF21 levels in response to the protein restriction, browning of sWAT was not induced in NZO mice and therefore not responsible for the increase in energy expenditure. The BAT mass gain (more than twofold) might instead account for this effect. Besides increasing BAT mass, the low-protein diets improved fat storage in white adipose tissue, which prevented ectopic hepatic fat accumulation, effects that were more pronounced in LP/HF than LP/HC mice.

An important finding is that protein restriction did not prevent hyperglycaemia and beta cell loss under conditions of a high-carbohydrate diet (LP/HC) despite increased FGF21 levels. The induction of hyperglycaemia was delayed by only 2 weeks in LP/HC mice in comparison to CON mice. To answer the question of why NZO mice on an LP/HC diet were not protected against the development of diabetes, several variables were compared between the LP/HC and LP/HF groups. Differences in body weight gain as a consequence of fat mass gain were not observed between LP/HC and LP/HF mice. Interestingly, the gain in lean mass was even higher in the LP/HC than the LP/HF mice. In addition, variations in energy expenditure could not explain the difference between the two groups. Energy expenditure was even higher in LP/HC mice in comparison to all the other mice at the end of the study. Thus, increased FGF21 and elevated energy expenditure do not protect against hyperglycaemia and diabetes per se. Both variables are not sufficient to prevent diabetes under conditions of high dietary carbohydrate.

Both low-protein-fed groups showed improved fat storage in brown and white adipose tissue, but liver and plasma triacylglycerols were significantly lowered only in LP/HF-treated mice compared with CON mice; LP/HC mice showed an intermediate state but no significant difference from CON mice. Analysis of liver ceramides revealed an increase in long-chain C22:0 and C24:0 ceramides in LP/HF mice, whereas LP/HC mice had similar concentrations of C22:0 and C24:0 ceramides to those of CON mice. In the liver, these ceramides (C22:0, C24:0), produced via ceramide synthase 2, have been shown to mediate protective effects on insulin sensitivity [24].

Moreover, defective glycogenolysis and gluconeogenesis regulation causes hyperglycaemia [25, 26]. Individuals with type 2 diabetes and NZO mice display an increased rate of gluconeogenesis [27–29]. In contrast to CON and LP/HC-fed mice, LP/HF mice showed the lowest hepatic glycogen content. Furthermore, FOXO1 phosphorylation was increased only in LP/HF mice by insulin. Phosphorylated FOXO1 is degraded and *Pepck* (also known as *Pck1*) transcription cannot be activated, which causes reduced hepatic glucose production [30]. The reduced rate of gluconeogenesis might therefore explain the divergence in blood glucose levels between LP/HC and LP/HF mice, and deserves further investigation. Interestingly, hepatic insulin sensitivity (Akt phosphorylation) was improved in LP/HC and LP/HF mice, which might be explained by FGF21 activation in these groups [31].

As mice on the LP/HC diet consumed nearly twice as much carbohydrate as CON mice and about 50% more than LP/HF-fed mice, we hypothesise that a high carbohydrate load together with a relatively high dietary fat content (33 kJ%) has severe glucolipotoxic effects that cannot be prevented by FGF21 and enhanced energy expenditure. Earlier studies showed the negative effects of a high-fat/high-carbohydrate diet on the islets of NZO mice. Under these glucolipotoxic conditions, beta cells lost GLUT2 and several important transcription factors, such as v-Maf musculoaponeurotic fibrosarcoma oncogene family, protein A (MAFA), pancreatic and duodenal homeobox 1 (PDX1) and NK6 homeobox 1 (NKX6.1), and underwent apoptosis, resulting in severe hyperglycaemia [16]. In fact, NZO mice on the LP/HF diet exhibited a tendency towards more and larger islets and a significantly elevated total pancreatic insulin concentration compared with CON and LP/HC mice. As recently shown, exogenous FGF21 treatment is sufficient to protect NZO mice from beta cell loss [11]. However, the actual data clearly demonstrate that this effect is compromised by high carbohydrate concentrations.

Dietary carbohydrate restriction reliably reduces high blood glucose and is the most effective therapy for diabetes, whereas dietary carbohydrates raise blood glucose levels [32, 33]. Individuals with type 2 diabetes benefit from substituting protein for carbohydrates [34], which reduces adiposity and associated disorders of metabolism by decreasing energy intake to some extent. A large number of studies, however, indicate that high-protein diets show no effects on fasting blood glucose, and that long-term high-protein/low-carbohydrate diets induce insulin resistance, increase the risk of type 2 diabetes, and are associated with increased mortality in humans [35, 36]. Paradoxically, low-protein/high-carbohydrate diets improve glucose tolerance and have the most beneficial effect on longevity in rodents without a reduction in total caloric intake [5, 37, 38]. Effects of low-protein diets on populations at high risk of diabetes remain unknown. Low-protein/high-carbohydrate diets are not optimal in periods of growth and reproduction during the early years and in elderly individuals (>65 years of age), but might be beneficial in terms of health and longevity in middle life (<65 years of age) [39]. This study demonstrates that protein restriction is only efficient in preventing hyperglycaemia when proteins are substituted by dietary fat instead of carbohydrates. The general induction of hyperphagia resulting from low protein intake [40] leads to an increased uptake of carbohydrates in the LP/HF group as well. When the uptake of carbohydrates is further increased (LP/HC), the protective effect of the low-protein-diet disappears. Thus, low-protein/high-carbohydrate diets might extend life only in animal models that are not diabetes-susceptible.

Taken together, the above experiments produce five notable conclusions. First, consistent with recent studies, we demonstrate the superior efficacy of dietary protein restriction in preventing the onset of diet-induced diabetes in male NZO mice. Second, this protective effect is not caused by hypophagia or body and fat mass loss, but rather by our third finding, an increase in energy expenditure due to increased BAT mass. Fourth, the protection against hyperglycaemia and diabetes depends on the dietary carbohydrate/fat ratio. Finally, a reduced rate of gluconeogenesis might protect against hyperglycaemia. Thus, the prevention of hyperglycaemia through protein restriction is compromised by high dietary carbohydrates despite increased FGF21 levels. It does not require body fat loss but increased hepatic long-chain ceramides, reduced gluconeogenesis and an elevated pancreatic islet mass.

## Electronic supplementary material

ESM (PDF 339 kb)

## Data Availability

The datasets generated during and/or analysed during the current study are available from the corresponding author on reasonable request.

## Abbreviations

BAT: Brown adipose tissue
CON: Control (diet/group)
DAG: Diacylglycerol
FGF21: Fibroblast growth factor 21
FOXO1: Forkhead box O1
gWAT: Gonadal white adipose tissue
LP/HC: Low-protein/high-carbohydrate (experimental diet/group)
LP/HF: Low-protein/high-fat (experimental diet/group)
NZO: New Zealand Obese
sWAT: Subcutaneous white adipose tissue
