# Supplementary material for: Dietary carbohydrates impair the protective effect of protein restriction against diabetes in NZO mice used as a model of type 2 diabetes
Source: Diabetologia. 2018 Mar 17;61(6):1459–69. doi: 10.1007/s00125-018-4595-1 (PMC6449005; doi:10.1007/s00125-018-4595-1)
Supplement: Supplementary file 1 — (PDF 339 kb) [file 125_2018_4595_MOESM1_ESM.pdf]

**ESM Table 1: Composition of diets**

| Product # | S8022-E122 | S8022-E120 | S8022-E121 |
|---|---|---|---|
| **Ingredient (g)** | **CON** | **LP/HC** | **LP/HF** |
| Casein | 200.00 | 50.00 | 50.00 |
| L-Cystine | 3.00 | 0.75 | 0.75 |
| Corn Starch | 264.00 | 412.50 | 320.50 |
| Maltodextrin 10 | 170.00 | 170.25 | 170.25 |
| Sucrose | 107.00 | 107.00 | 107.00 |
| Cellulose | 50.00 | 50.00 | 50.00 |
| Soybean Oil | 25.00 | 25.00 | 25.00 |
| Lard | 133.50 | 135.00 | 227.00 |
| Mineral Mix S10022G | 35.00 | 35.00 | 35.00 |
| Calcium Phosphate, Dibasic | 0 | 0.50 | 0.50 |
| Potassium Phosphate, Monobasic | 0 | 1.50 | 1.50 |
| Vitamin Mix V10037 | 10.00 | 10.00 | 10.00 |
| Choline Bitrartrate | 2.50 | 2.50 | 2.50 |
| Sum (g) | 1000 | 1000 | 1000 |
| kJ/gram | 17.99 | 18.41 | 20.08 |
| | | | |
| Protein (gm%) | 18 | 4 | 4 |
| Carbohydrate (gm%) | 55 | 69 | 60 |
| Fat (gm%) | 16 | 16 | 25 |
| | | | |
| Protein (kJ%) | 16 | 4 | 4 |
| Carbohydrate (kJ%) | 51 | 63 | 49 |
| Fat (kJ%) | 33 | 33 | 47 |

**ESM Table 2: Antibodies and ELISA**

| Antibody | Product # | Company | Dilution |
|---|---|---|---|
| **western immunoblot analysis** | | | |
| protein kinase B (PKB/Akt) | 9272 | Cell Signaling | 1:1,000 |
| phospho-Akt $Ser^{473}$ | 9271 | Cell Signaling | 1:1,000 |
| FoxO1 | 2880 | Cell Signaling | 1:1,000 |
| phospho-FoxO1 $Thr^{24}$ | 9464S | Cell Signaling | 1:1,000 |
| GAPDH | AM4300 | ThermoFisher | 1:25,000 |
| α-Tubulin | T6074 | Sigma | 1:1,000 |
| HRP-conjugated anti-mouse and anti-rabbit IgG | 315-035-008 | Dianova | 1:20,000 |
| DyLightTM 680 conjugated anti-mouse IgG | 35519 | Thermo Scientific | 1:15,000 |
| DyLightTM800 4X-PEG anti-rabbit | SA5-35571 | Thermo Scientific | 1:15,000 |
| **immunohistochemistry of pancreatic islets** | | | |
| mouse monoclonal anti-insulin | J2018 | Sigma-Aldrich | 1:250,000 |
| Biotin-labelled donkey anti-mouse antibody | 715-065-150 | Dianova | 1:800 |
| biotin-streptABC complex | Vectastain ABC kit PK-6100 | Vector Lab. | |
| **ELISA** | | | |
| Insulin | 80-INSMSU-E01 | ALPCO Diagnostics | |
| Mouse Adiponectin/Acrp30 DuoSet ELISA | DY1119 | R&D Systems GmbH | |
| Mouse and Rat FGF-21 ELISA | RD291108200R | BioVendor | |

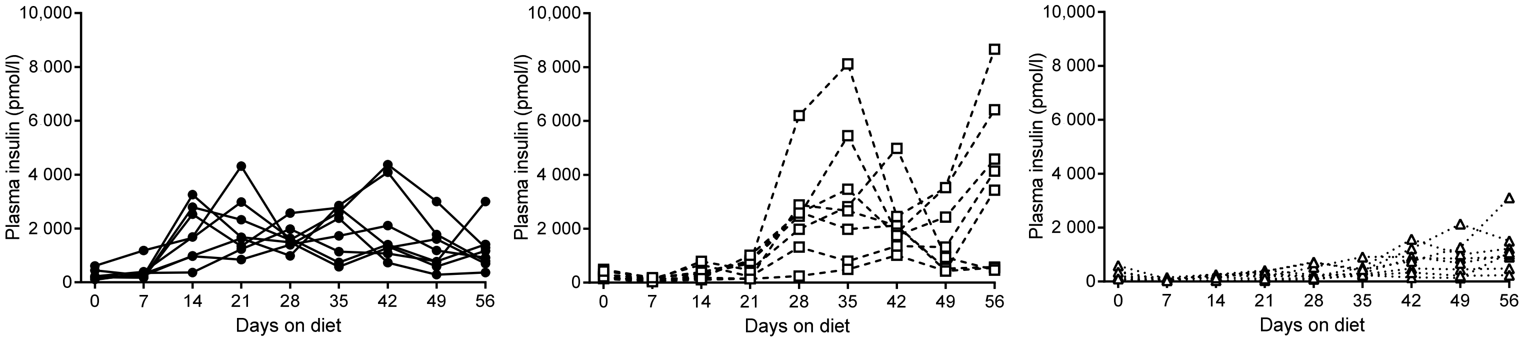


**ESM Fig. 1** Mice were treated as described in Fig. 1. At the age of 3 weeks, NZO mice were placed on a control diet (CON) for 1 week, at which point a random subgroup of animals was transferred to LP/HC or LP/HF diet for 8 weeks. Individual random plasma insulin concentrations. Black circles, CON; white squares, LP/HC; white triangles, LP/HF.

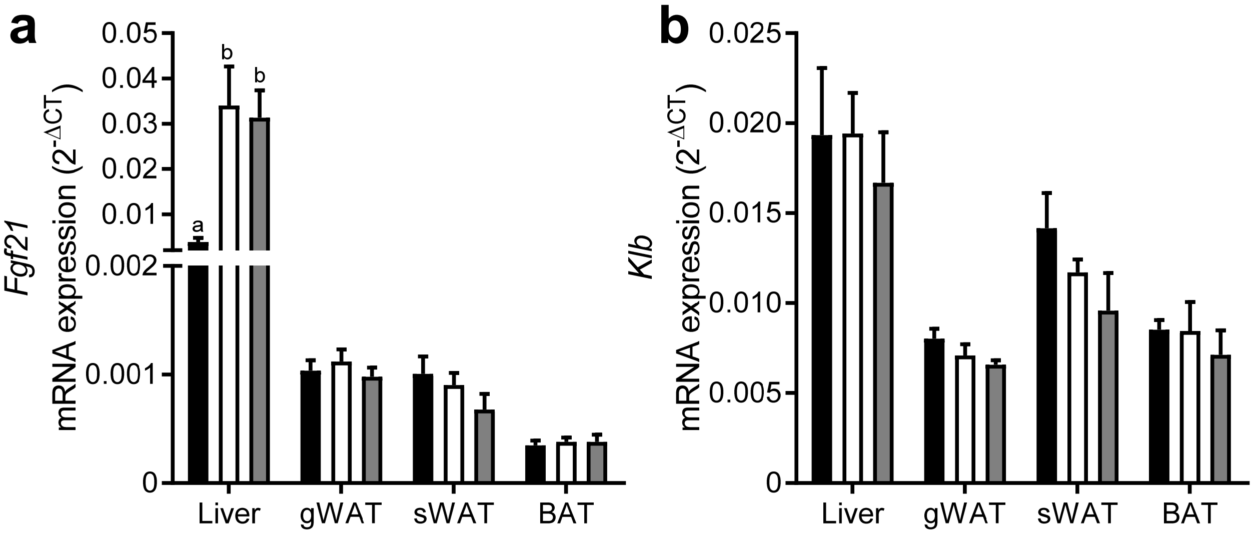


**ESM Fig. 2** Effects of protein restriction on *Fgf21* and *Klb* mRNA expression. Tissues were collected from mice which were treated as described in Fig. 1. Eight weeks after the diet switch, 6-hour fasted animals were sacrificed. Gene expression of (**a**) *Fgf21* and (**b**) *Klb* in indicated tissues were measured via real-time PCR. Black bars, CON; white bars, LP/HC; grey bars, LP/HF. Data are presented as mean ± SEM ($n$=8/group). Differences between groups were calculated by one-way ANOVA. Mean values with different lowercase letters differ with $p$<0.05.

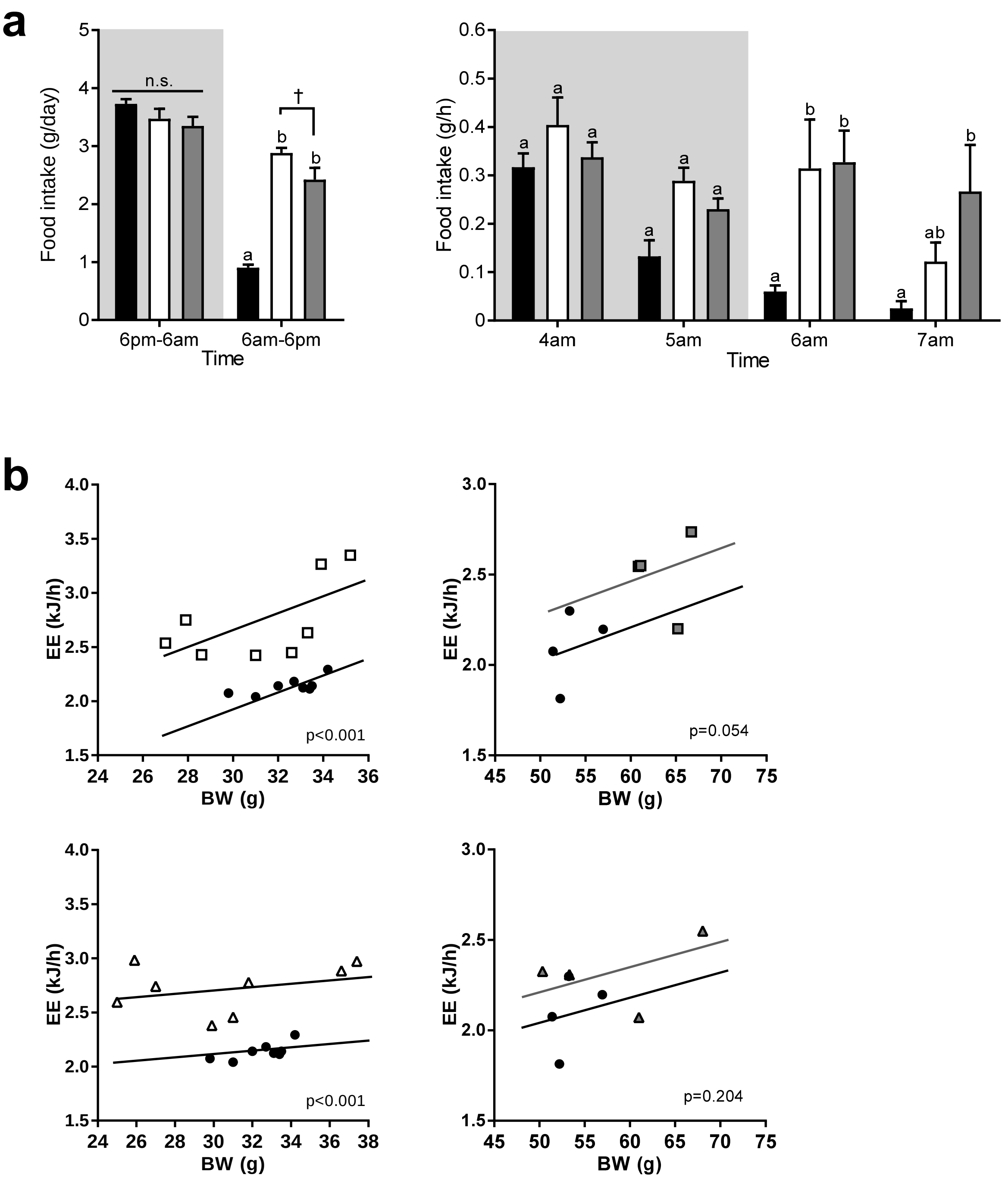


**ESM Fig. 3** Protein restriction increases energy intake and energy expenditure in NZO mice. Mice were treated as described in Fig. 1. (**a**) Average food intake for indicated periods throughout the first week after diet switch. (**b**) Scatter plots illustrating the relationship between body weight and energy expenditure (EE), based on ANCOVA, in NZO mice fed control or different low-protein diets for one (left panel) or eight (right panel) weeks. Black bars and circles, CON; white bars and squares, LP/HC; grey bars and triangles, LP/HF. Data are presented as mean ± SEM (week 1: $n$=8/group; week 8: $n$=4/group). Differences between groups were calculated by one-way ANOVA (**a**). Mean values with different lowercase letters differ with $p<0.05$. $^{\dagger}0.1>p>0.05$.

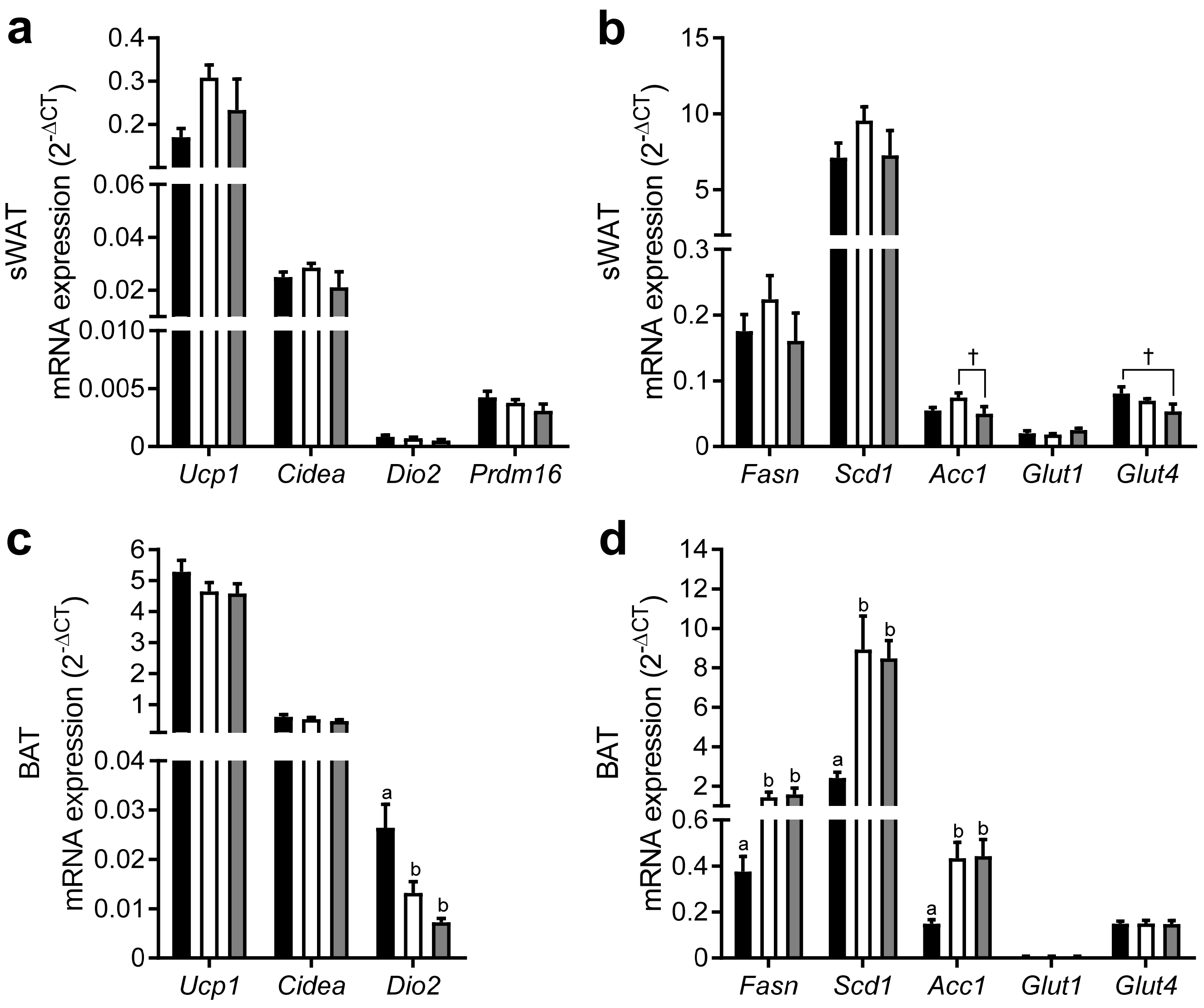


**ESM Fig. 4** Effects of protein restriction on gene expression in fat depots. Tissues were collected from mice which were treated as described in Fig. 1. Eight weeks after the diet switch, 6-hour fasted animals were sacrificed. Thermogenic genes from (**a**) sWAT and (**c**) BAT and lipogenic and glucose transporter genes from (**b**) sWAT and ( **d**) BAT were measured via real-time PCR. Black bars, CON; white bars, LP/HC; grey bars, LP/HF. Data are presented as mean ± SEM (*n*=8/group). Differences between groups were calculated by one-way ANOVA. Mean values with different lowercase letters differ with $p<0.05$. [†]$0.1>p>0.05$.

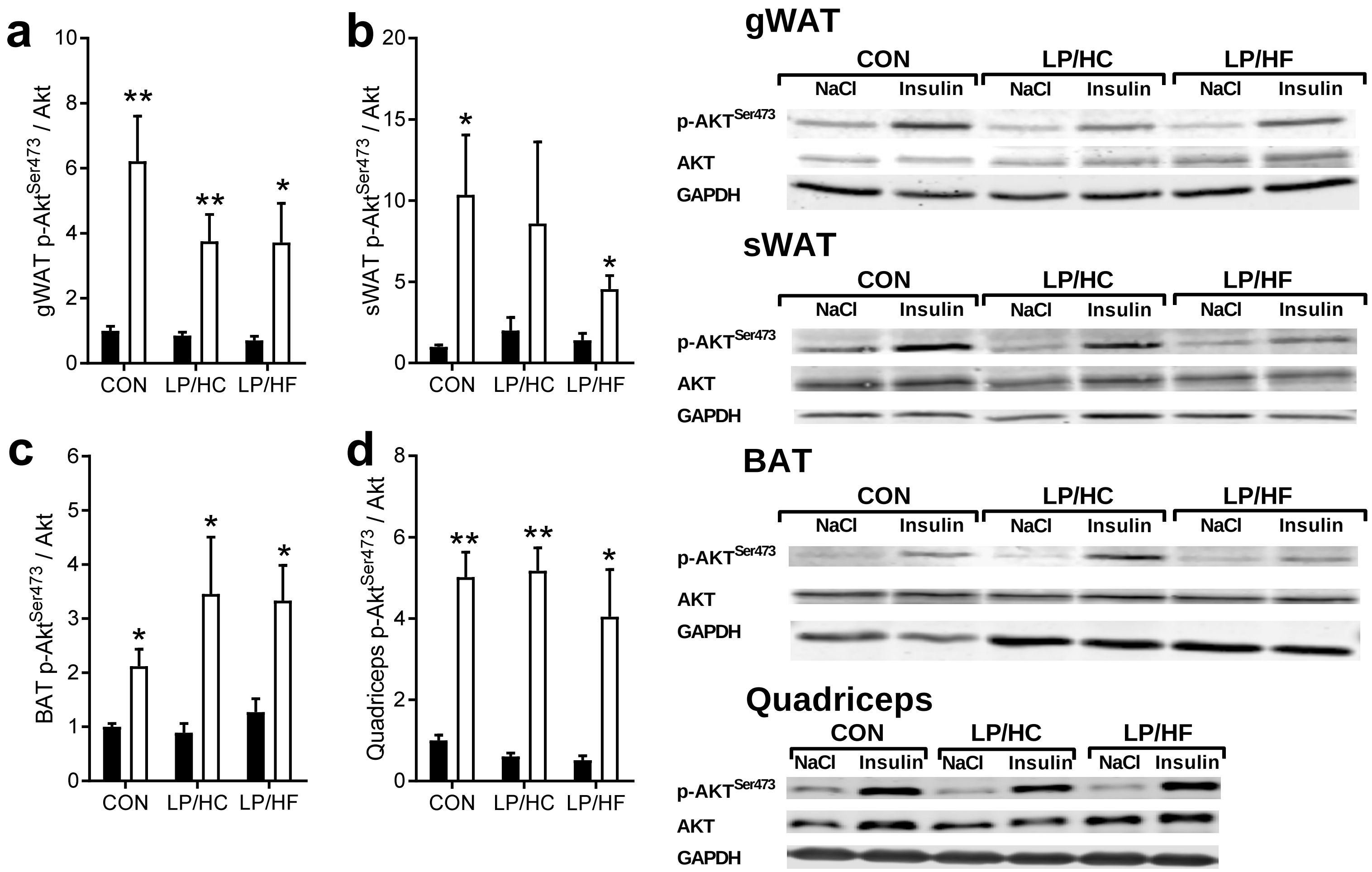


**ESM Fig. 5** Effects of dietary protein restriction on insulin sensitivity in NZO mice. Mice were treated as described in Fig. 1. Eight weeks after the diet switch, 6-hour fasted animals were treated subcutaneously with NaCl or insulin (7 IU/BWkg) 15 min before sacrifice. Western blots of total and phosphorylated Akt in (**a**) gWAT, (**b**) sWAT, (**c**) BAT, and (**d**) quadriceps muscle. Black bars, NaCl; white bars, Insulin. Data are presented as mean ± SEM (*n*=6 per group). Differences between groups were analysed using two-tailed *t*-test. *$p<0.05$, **$p<0.01$.
